# Prevalence and antimicrobial susceptibility pattern of *Salmonella* and *Shigella* species among asymptomatic food handlers working in Haramaya University cafeterias, Eastern Ethiopia

**DOI:** 10.1186/s13104-018-3189-9

**Published:** 2018-01-25

**Authors:** Dadi Marami, Konjit Hailu, Moti Tolera

**Affiliations:** 1 Department of Medical Laboratory Sciences, College of Health and Medical Sciences, Haramaya University, P.O. Box: 235, Harar, Ethiopia; 2 Haramaya University Higher Health Center, Haramaya University, P.O. Box: 138, Dire Dawa, Ethiopia; 3 School of Public Health, College of Health and Medical Sciences, Haramaya University, P.O. Box: 235, Harar, Ethiopia

**Keywords:** *Salmonella* species, *Shigella* species, Antimicrobial resistance, Food handler, University cafeteria

## Abstract

**Objective:**

Salmonellosis and Shigellosis remain a major public health problem across the globe, particularly in developing countries like Ethiopia, where hand hygiene and food microbiology are still below the required standards. The growing problem of antimicrobial resistance species also continues to pose public health challenges. This study assessed the prevalence and antimicrobial susceptibility pattern of *Salmonella* and *Shigella* species among asymptomatic food handlers. A cross-sectional study was conducted among 417 randomly selected asymptomatic food handlers. Data were collected using a structured questionnaire. The stool specimens collected were examined for *Salmonella* and *Shigella* species using standard bacteriological methods. Descriptive statistics were used to describe the basic features of the data.

**Results:**

The overall prevalence of *Salmonella* and *Shigella* species was 5.04%. *Salmonella* and *Shigella* species were 76.2% resistant to both co-trimoxazole and tetracycline, 71.4% to amoxicillin and 66.7% to chloramphenicol. Moreover, 85.7% of *Salmonella* and *Shigella* species were multidrug resistant. The findings highlighted the food handlers as potential sources of food borne infections, which demands the establishment of appropriate hygiene and sanitary control measures at the University cafeterias.

## Introduction

*Salmonella* and *Shigella* species are the most common causes of food and water-borne gastroenteritis in humans, which remains an important health problem worldwide [1, 2]. According to World Health Organization (WHO) estimates, there are about 16 million new cases and 600,000 deaths from typhoid fever each year worldwide [2]. *Shigella* strains have also continued to play a major role in the etiology of inflammatory diarrhea and dysentery [3].

The emergence of antimicrobial resistant *Salmonella* and *Shigella* spp. are other global challenges, especially in developing countries where there is an increased misuse of antimicrobial agents in humans and animals [2, 4]. An example of the global threat of antimicrobial resistant *Salmonella* spp. have been widely reported in Europe and America [5]. In Ethiopia, high frequency of resistant *Salmonella* and *Shigella* spp. have been observed among the following antimicrobial groups: tetracycline (52.5%, 82.4%), co-trimoxazole (37.5%, 76.5%) and ampicillin (60%, 47.1%), respectively [6].

*Salmonella* and *Shigella* spp. are commonly transmitted through the feco-oral route and close contact with infected individuals [7]. Asymptomatic food handlers are known to play crucial role in transmitting the infections and continue to pose a threat to public health [4, 8]. Previous reports indicate that food prepared in large quantities by the involvement of a large number of food handlers at higher learning institutions has been often prone to contamination by infected or asymptomatic carriers of infections leading to outbreaks of food borne diseases [9].

In addition, isolation of *Shigella* and *Salmonella* spp. in most parts of African laboratories, including Ethiopia remain a challenge, due to inadequate laboratory facilities to allow accurate detection and performance of antimicrobial susceptibility testing [10]. As a result, information relating to *Salmonella*, *Shigella* spp. and their antimicrobial susceptibility patterns in Ethiopia are scarce. Thus, this study was aimed to determine the prevalence and antimicrobial susceptibility patterns of *Salmonella* and *Shigella* spp. among food handlers working at Haramaya University cafeterias.

## Main text

### Methods

#### Study setting

A cross-sectional study was conducted among asymptomatic food handlers working at Haramaya University, Eastern Ethiopia from August 2015 to January 2016. The University is located at a distance of 510 km from Addis Ababa. Currently, the University cafeterias serve meals for 30,000 students and staffs.

#### Sample size and sampling technique

The sample size was calculated using a single population proportion formula by taking an estimated 50% proportion (p = 0.5) of *Salmonella* and *Shigella* spp., 5% margin of error (d = 0.05) and 95% confidence interval (z = 1.96). The initial sample size was 384, and by considering 10% non-response rate, the final sample size was determined to be 422. To select representative participants, the final sample size was proportionally allocated to each stratum, and food handlers were selected using systematic random sampling technique. Participants who reported to have never used any antimicrobial in the last 2 weeks and during the study period were included in the study. A complete list of food handlers was obtained from the human resource management directorate of Haramaya University.

#### Data collection procedure and sample collection

A pre-tested structured questionnaire was used to collect data relating to sociodemographic characteristics of the study participants. The questionnaire was developed from validated tools [4, 11, 12].

The stool specimens were collected after brief instruction in sterile, leakproof test tubes containing a Cary-Blair transporting media (CM0519B, Oxoid, Ltd, UK), and transported in the temperature controlled cold box to the microbiology laboratory unit of the College of Health and Medical Sciences for bacteriologic analysis.

#### Culture isolation and characterization

Culture isolation and identification of *Salmonella* and *Shigella* spp. was performed based on the standard procedure [13]. Briefly: 25 g of stool specimen was homogenized in 225 ml of buffered peptone water (CM1049B, Oxoid, Ltd, UK) using blender (Stomacher 400, Seward, England) for 1 min. A volume of 1 ml aliquot was transferred into 10 ml of Selenite F broth (CM0399B, Oxoid, Ltd, UK) and incubated at 37 °C. After overnight incubation, a loopful of culture was taken and streaked on Xylose lysine deoxycholate agar plate (TV5028N, Oxoid, Ltd, UK) and incubated overnight at 37 °C. The culture plates were examined for the presence of *Salmonella* spp. (Pink–red with a black center colonies) and *Shigella* spp. (Pink-red colonies) [13, 14]. Culture positives were characterized by standard biochemical tests, including motility (CM0435B, Oxoid, Ltd, UK), indole test (CM0967B, Oxoid, Ltd, UK), and Kligler iron agar reactions (CM0033B, Oxoid, Ltd, UK). The result of each culture was read after incubation for 24–48 h at 37 °C [15, 16]. The morphology of the isolate was also characterized microscopically using the Gram staining technique [15].

#### Antimicrobial susceptibility testing

The antimicrobial susceptibility test was done using the modified disk diffusion technique on Mueller–Hinton agar (PO5007A, Oxoid, Ltd, UK) in accordance with the Clinical and Laboratory Standards Institute (CLSI) guideline [17]. In brief, about 3–5 colonies of the same type were picked up from culture media and mixed with 5 ml of sterile normal saline and standardized to 0.5 McFarland. A sterile cotton swab was used to distribute the bacterial suspension evenly over the entire surface of the Mueller–Hinton agar plates. Then, antimicrobial disks were applied to the surface of the inoculated plates using an automatic disk dispenser (ST6090, Oxoid, Ltd, UK). Nine antimicrobial disks (Oxoid, Ltd, UK) including ampicillin (10 mg), tetracycline (30 mg), chloramphenicol (30 mg), gentamicin (10 mg), ciprofloxacin (10 mg), co-trimoxazole (1.25/23.75 µg), ceftazidime (30 µg), norfloxacin (10 mg) and ceftriaxone (30 µg) were applied on the culture plates. The zone of inhibition was read after 24 h of incubation at 37 °C [17]. Multidrug resistance (MDR) is defined when isolates are resistant to two or more antimicrobials of different group [18].

#### Quality control

The questionnaire was first prepared in English and translated into two local languages (*Amharic* and *Afan Oromo*) and then translated back to English by different bilingual experts to check the consistency. The questionnaire was reviewed by Medical Microbiologists, and pre-tested on 5% of the food handlers working in Dire Dawa University cafeterias to check the practicability and the applicability of the questionnaire.

Data collectors and supervisors were trained for 2 days on the objective of the study, interviewing techniques, and data quality management. Regular supervision, spot checking and reviewing the completeness and consistency of questionnaires on a daily basis were made to assure the quality of data.

The quality of each new batch of culture medium and antimicrobial disks was checked by testing *E. coli* (ATCC^®^ 25922), *S. aureus* (ATCC^®^ 25923), and *P. aeruginosa* (ATCC^®^ 27853) reference strains. All testing results obtained from the reference strains were within the established quality control limits of the CLSI guideline [17]. The result of the culture and antimicrobial tests was read by two Medical Microbiologists.

#### Methods of data analysis

The data were checked for completeness, cleaned and double entered into Epi-Info version 3.5.1 (CDC, Atlanta, GA, USA). The two entries were compared, and discrepancies were resolved by referring to the original document and exported to the Statistical Package for Social Sciences (SPSS) version 20 (Inc, Chicago, IL) for analysis. Results were presented as percentages, mean, and standard deviation.

### Results

#### Socio-demographic characteristics

A total of 417 asymptomatic food handlers were enrolled in this study, making a response rate of 98.8%. Of these, 79.4% were female; with a male to female ratio of 0.26:1. The majority of participants age was between 31 and 40 years (39.3%) with the mean age of 36.1 (Standard deviation ± 8.7 years). Up to 42.4% were literate with primary level education (1–8th grade). The majority of the participants was currently married and served as a food handler for more than 5 years (58.5%) and (66.2%), respectively (Table 1).

**Table 1:** The prevalence of *Salmonella* and *Shigella* spp. in respective to socio-demographic characteristics of asymptomatic food handlers working in Haramaya University cafeterias, Eastern Ethiopia from August 2015 to January 2016

Socio-demographic characteristics	*Salmonella* and *Shigella* spp. (%)	
	Pos. (%)	Neg. (%)
Gender		
Female	16 (76.2)	315 (79.5)
Male	5 (23.8)	81 (20.5)
Age group (in years)		
> 40	11 (52.4)	134 (33.8)
31–40	7 (33.3)	157 (39.6)
21–30	2 (9.5)	89 (22.5)
≤ 20	1 (4.8)	16 (4)
Educational status		
No formal education	8 (38.1)	91 (23)
Primary level (1–8th)	6 (28.6)	174 (43.9)
Secondary level (9–12th)	4 (19)	89 (22.5)
Tertiary level (> 12th)	3 (14.3)	42 (10.6)
Current marital status		
In marriage	12 (57.1)	229 (57.8)
Divorced	2 (9.5)	59 (14.9)
Widowed	4 (19)	37 (9.3)
Unmarried	3 (14.3)	71 (17.9)
Year of service (in year)		
More than 5	13 (61.9)	263 (66.4)
≤ 5	8 (38.1)	133 (33.6)

#### Prevalence of *Salmonella* and *Shigella* spp.

The overall prevalence of *Salmonella* and *Shigella* spp. was 5.04%. Of these, 3.6% and 1.4% were *Salmonella* and *Shigella* spp., respectively. The most frequently isolated *Salmonella* spp. was *S. typhi* (2.2%) followed by *S. paratyphi* (1%).

*Salmonella* and *Shigella* spp. were more prevalent among females (76.2%), age more than 40 years (52.4%), had no formal education (38.1%), were married (57.1%) and had been working at the cafeteria for more than 5 years (61.9%) (Table 1).

#### Antimicrobial susceptibility pattern

More than 85% of *Salmonella* and *Shigella* isolates were sensitive to both Ceftazidime and Ciprofloxacin, and 81% to both Ceftriaxone and Norfloxacin. A higher rate of resistance (76.2%) was observed to both Co-trimoxazole and Tetracycline, 71.4% to Ampicillin and 66.7% to Chloramphenicol (Table 2).

**Table 2:** Antimicrobial susceptibility pattern of *Salmonella* and *Shigella* spp. isolated from stool specimens of asymptomatic food handlers working in Haramaya University cafeterias, Eastern Ethiopia from August 2015 to January 2016

Bacterial isolates	Total isolates	Antimicrobial susceptibility (n %)									
		Pattern	AM	CRO	CAZ	CHL	CIP	COT	GN	NOR	TE
*S. typhi*	9	S	2 (22.2)	7 (77.8)	9 (100)	1 (11.1)	6 (66.7)	1 (11.1)	7 (77.8)	8 (88.9)	1 (11.1)
		I	0	0	0	0	0	0	0	0	0
		R	7 (77.8)	2 (22.2)	0	8 (88.9)	3 (33.3)	8 (88.9)	3 (22.2)	1 (11.1)	8 (88.9)
*S. paratyphi*	4	S	0	3 (75)	3 (75)	0	4 (100)	1 (25)	2 (50)	2 (50)	0
		I	0	0	1 (25)	2 (50)	0	0	0	0	2 (50)
		R	4 (100)	1 (25)	0	2 (50)	0	3 (75)	2 (50)	2 (50)	2 (50)
Other *Salmonella* spp.	2	S	0	2 (100)	2 (100)	0	2 (100)	1 (50)	2 (100)	2 (100)	1 (50)
		I	0	0	0	1 (50)	0	0	0	0	0
		R	2 (100)	0	0	1 (50)	0	1 (50)	0	0	1 (50)
*Shigella* spp.	6	S	4 (66.7)	5 (83.3)	4 (66.7)	2 (33.3)	6 (100)	2 (33.3)	4 (66.7)	5 (83.3)	1 (16.7)
		I	0	0	1 (16.7)	1 (16.7)	0	0	0	0	0
		R	2 (33.3)	1 (16.7)	1 (16.7)	3 (50)	0	4 (66.7)	2 (33.3)	1 (16.7)	5 (83.3)
Total	21	S	6 (28.6)	17 (81)	18 (85.7)	3 (14.3)	18 (85.7)	5 (23.8	15 (71.4)	17 (81)	3 (14.3)
		I	0	0	2 (9.5)	4 (19)	0	0	0	0	2 (9.5)
		R	15 (71.4)	4 (19)	1 (4.8)	14 (66.7)	3 (14.3)	16 (76.2)	6 (28.6)	4 (19)	16 (76.2)

*S* sensitive, *I* intermediate, *R* resistance, *AM* ampicillin, *CRO* ceftriaxone, *CAZ* ceftazidime, *CHL* chloramphenicol, *CIP* ciprofloxacin, *COT* cotrimoxazole, *CN* gentamicin, *NOR* norfloxacin, *TE* tetracycline

*Salmonella typhi* was highly sensitive to ceftazidime (100%), norfloxacin (88.9%), 77.8% to both ceftriaxone and gentamicin; whereas there was 88.9% resistance to chloramphenicol, co-trimoxazole and tetracycline. On the other hand, *Shigella* species were 100% sensitive to ciprofloxacin and 83.3% sensitive to both ceftriaxone and norfloxacin; while resistance to tetracycline was 83.3% and co-trimoxazole was 66.7% (Table 2).

#### Multidrug resistance pattern

The overall multidrug resistance *Salmonella* and *Shigella* spp. was 85.7%. Of this, 88.9% was *Salmonella* spp. Among *Salmonella* spp., the most frequent MDR isolates were *S. typhi* (50%) (Table 3).

**Table 3:** Multidrug resistance pattern of *Salmonella* and *Shigella* spp. isolated from stool of asymptomatic food handlers working in Haramaya University cafeterias, Eastern Ethiopia from August 2015 to January 2016

MDR pattern	*S. typhi* (n = 9)	*S. paratyphi* (n = 5)	Other spp. (n = 2)	*Shigella* (n = 2)
AM, COT	1 (11.1)	0	0	0
COT, TE	3 (33.3)	2 (40)	0	0
CHL, TE	0	1 (20)	0	1 (50)
AM, CHL, COT	1 (11.1)	1 (20)	1 (50)	0
CHL, COT, TE	2 (22.2)	1 (20)	1 (50)	0
AM, CHL, COT, TE	2 (22.2)	0	0	1 (10)
MDR by spp.	9 (50)	5 (27.8)	2 (11.1)	2 (11.1)
MDR by genera (N = 18)	16 (88.9)			2 (11.1)
Overall MDR (N = 21)	18 (85.71)			

*n* number of isolates, *N* total number of isolates

### Discussion

In this study, the overall prevalence of *S. typhi* was 3.6%. The finding is comparable to a study conducted in Addis Ababa University, Ethiopia (3.4%) [19]; but relatively higher than reports from other parts of Ethiopia, such as Dilla University (0.93%) [8] and Mekelle University (1%) [12]. However, it is lower compared with a study done in Arba Minch University, Southern Ethiopia (6.9%) [20] and Bahir Dar, Ethiopia (80%) [4]. On the other hand, a higher rate of *Shigella* spp. (1.4%) was isolated in this study. This is much more than an expected for the occurrence of a bacillary dysentery outbreak. However, the prevalence is lower compared to reports elsewhere from Ethiopia such as in Gondar University (2.7%) [11] and Arba Minch University (3%) [20]. The possible explanation for this variation might be due to differences in the sample size (small sample size might overestimate the proportion), geographical variation and socioeconomic conditions.

*Salmonella typhi* showed a high sensitivity to ceftazidime (100%), norfloxacin (88.9%), ceftriaxone (77.8%) and norfloxacin (66.7%); whereas there was 88.9% resistance to chloramphenicol, tetracycline and co-trimoxazole. This is comparable to studies conducted in other parts of Ethiopia such as in Mekelle University, where *S. typhi* showed 100% sensitivity to norfloxacin and 75% resistance to ampicillin, tetracycline and chloramphenicol [12] and in a Gonder University in which there was 100% sensitive to norfloxacin and 50% resistance to both tetracycline and co-trimoxazole [11], indicating that antimicrobial resistance of *S. typhi* is an increasing concern.

In the current study, *Shigella* spp. were 100% sensitive to ciprofloxacin, 83.3% to both ceftriaxone and norfloxacin; while 83.3% were resistant to tetracycline. This is consistent with reports from Gonder University, Ethiopia, where *Shigella* species showed high level of sensitivity to ciprofloxacin (100%) and norfloxacin (87.5%), but with 75% resistance to tetracycline [11]. The similarity in the antimicrobial susceptibility pattern among *Shigella* spp. may be due to the availability and unrestricted use of antimicrobials [18].

This study also showed high MDR (85.7%) *Salmonella* and *Shigella* spp., where MDR was higher among the *Salmonella* spp. (88.9%), and less among the *Shigella* spp. (11.1%). The MDR *Salmonella* spp. was low compared to a report from Addis Ababa University, Ethiopia (100%) [22] but higher compared to a study conducted in Gonder University (46.2%) [13]. On the other hand, the MDR *Shigella* spp. is also low compared to studies conducted in other parts of Ethiopia such as Addis Ababa University (100%) [21] and Arba Minch University (100%) [14]. The cause of variations in the prevalence of MDR is unknown, but might be due to inappropriate empirical antimicrobial treatment, easy availability and indiscriminate use of common antimicrobials.

The study indicates a higher prevalence of *Salmonella* and *Shigella* spp. among food handlers who had more than 5 years’ work experience (61.9%) compared to less than or equal to 5 years (38.1%). This is in line with a study done in Arba Minch University, Ethiopia (32.4%) [20]. However, the results are lower compared to a study done in Mekelle University, Ethiopia in which 60% of food handlers who served for less than 5 years were infected [12]. Lack of regular medical checkups, food safety training, inadequate supervision and a low level of literacy of food handlers might contribute to this difference. The prevalence of antimicrobial resistant *Salmonella* and *Shigella* spp. in this study is high. Infected or asymptomatic carriers who are handling food on a daily basis can act as sources of infection to consumers via the food chain. A periodic medical check-up program with intensive health education could improve the workers’ health status.

### Limitations

Fingernail content examination, which might support the idea of contamination due to poor food handling practices, was not performed. In spite of this limitation, the methods used to isolate and characterize the antimicrobial susceptibility pattern of *Salmonella* and *Shigella* spp. are comprehensive.

## Abbreviations

CLSI: Clinical and Laboratory Standards Institute
MDR: Multidrug Resistance
WHO: World Health Organization

## References

[1] Senthilkumar B, Prabakaran G (2005). Multidrug resistant *Salmonella typhi* in asymptomatic typhoid carriers among food handlers in Namakkal district, Tamil Nadu. Indian J Med Microbiol..

[2] World Health Organization (2008). Foodborne disease outbreaks: guidelines for investigation and control.

[3] Kansakar P, Malla S, Ghimire R (2007). *Shigella* isolates of Nepal: changes in the incidence of *Shigella* subgroups and trends of antimicrobial susceptibility pattern. Kuthmadu Univ Med J..

[4] Bayeh A, Fantahun B, Bezabih B (2010). Prevalence of *Salmonella typhi* and intestinal parasites among food handlers in Bahir Dar Town, Northwest Ethiopia. Ethiop J Heal Dev..

[5] Hendriksen R, Mikoleit M, Kornschober C, Rickert R, Duyne S, Kjelso C, Hasman H, Cormican M, Mevius D, Threlfall J, Angulo F, Aarestrup F (2009). Multidrug resistant *Salmonella* concord infections in Europe and the United States in children adopted from Ethiopia. Pediatr Infect Dis J..

[6] Getachew M, Gebru M, Tsehaynesh L, Abraham A (2014). Prevalence and antimicrobial susceptibility patterns of *Salmonella* serovars and *Shigella* species in Butajira, central Ethiopia. J Microb Biochem Technol..

[7] Gashaw A, Afework K, Feleke M, Moges T, Kahsay H (2008). Prevalence of bacteria and intestinal parasites among food-handlers in Gondar Town, Northwest Ethiopia. J Health Popul Nutr..

[8] Misganaw B, David W (2013). A study of *Salmonella* carriage among asymptomatic food-handlers in southern Ethiopia. Int J Nutr Food Sci..

[9] Conradie N (2007). Small and micro enterprises—aspects of knowledge, attitudes and practices of managers’ and food handlers’ knowledge of food safety in the proximity of Tygerberg academic hospital, Western Cape. SAJCN..

[10] Sosa A, Byarugaba D, Amabile-Cuevas C, Hsueh P-R, Kariuki S, Okeke I (2010). Antimicrobial resistance in developing countries.

[11] Mulat D, Moges T, Feleke M, Mucheye G (2013). Infectious diseases & therapy bacterial profile and antimicrobial susceptibility pattern among food handlers at Gondar University cafeteria, Northwest Ethiopia. J Infect Dis Ther..

[12] Araya G, Kelemework A, Letemichael N, Tsehaye A, Shwaye B, Megbaru A, Muthupandian S (2014). Prevalence of *Salmonella typhi* and intestinal parasites among food handlers in Mekelle University student cafeteria, Mekelle, Ethiopia. Food Control.

[13] Legesse G, Nishanwork W, Amsalu F (2014). Identification of drug-resistant *Salmonella* from food handlers at the University of Gondar, Ethiopia. BMC Res Notes..

[14] Getenet B, Haimanot T (2014). Prevalence of intestinal parasite, *Shigella* and *Salmonella* species among diarrheal children in Jimma health center, Jimma Southwest Ethiopia. Ann Clin Microbiol Antimicrob..

[15] Cheesbrough M (2006). District laboratory practice in tropical countries, part 2.

[16] Perilla M (2003). Bacterial agents of enteric diseases of public health concern. Manual for the laboratory identification and antimicrobial susceptibility testing of bacterial pathogens of public health importance in the developing World.

[17] Clinical and Laboratory Standards Institute (2015). Performance standards for antimicrobial susceptibility testing; twenty-fifth informational supplement. CLSI document M100-S25.

[18] Asrat D (2008). *Shigella* and *Salmonella* serogroups and their antibiotic susceptibility patterns in Ethiopia. East Mediterr Health J..

[19] Fentabil G, Solomon G, Haile A, Tesfu K, Nigatu K (2014). Prevalence and antimicrobial resistance of *Salmonella* isolated from food handlers in Addis Ababa University students’ cafeteria, Ethiopia. Afr J Basic Appl Sci.

[20] Mama M, Getaneh A (2016). Prevalence, antimicrobial susceptibility patterns and associated risk factors of *Shigella* and *Salmonella* among food handlers in Arba Minch University, South Ethiopia. BMC Infect Dis.

[21] Addis A, Daniel K, Mekonnen D, Negatu T, Saba G, Seyfe Z, Kassu D, Gebru M, Yeshiwodim M, Mohammedaman M (2015). Prevalence of intestinal parasites, *Salmonella* and *Shigella* among apparently health food handlers of Addis Ababa University student’s cafeteria, Addis Ababa, Ethiopia. BMC Res Notes..

